# Cardiac Laterality: Surgical Results of Right Atrial Isomerism

**DOI:** 10.3390/diseases11040170

**Published:** 2023-11-20

**Authors:** Diego B. Ortega-Zhindón, Nonanzit Pérez-Hernández, José Manuel Rodríguez-Pérez, José A. García-Montes, Juan Calderón-Colmenero, Frida Rivera-Buendía, Jorge L. Cervantes-Salazar

**Affiliations:** 1 Department of Pediatric Cardiac Surgery and Congenital Heart Disease, Instituto Nacional de Cardiología Ignacio Chávez, Mexico City 14080, Mexico; diegob.ortegaz@gmail.com; 2 Department of Molecular Biology, Instituto Nacional de Cardiología Ignacio Chávez, Mexico City 14080, Mexico; unicanona@yahoo.com.mx (N.P.-H.); josemanuel_rodriguezperez@yahoo.com.mx (J.M.R.-P.); 3 Department of Interventional Cardiology in Congenital Heart Disease, Instituto Nacional de Cardiología Ignacio Chávez, Mexico City 14080, Mexico; pepegamon@yahoo.com.mx; 4 Department of Pediatric Cardiology, Instituto Nacional de Cardiología Ignacio Chávez, Mexico City 14080, Mexico; juanecalderon@yahoo.com.mx; 5 Department of Clinical Research, Instituto Nacional de Cardiología Ignacio Chávez, Mexico City 14080, Mexico; frida.rivera06@gmail.com

**Keywords:** atrial isomerism, right atrial isomerism, single ventricle, cardiac surgery, congenital heart disease

## Abstract

Right atrial isomerism (RAI) is a complex entity with varying diagnostic and treatment outcomes due to its rarity. Treatment options range from palliative to corrective surgeries, resulting in heterogeneous outcomes. The aim of this study was to analyze the results obtained after cardiac surgery in patients with RAI. A retrospective study was conducted, including patients diagnosed with RAI who underwent cardiac surgery. Their follow-up was from 1 January 2010 to 31 March 2020. Demographic characteristics and perioperative conditions were described. Thirty-eight patients were included, the median age was 4 years (IQR 2–9.2) and 57.9% were men. The main diagnoses were atrioventricular canal (63.2%) and pulmonary stenosis (55.3%). The most common surgical procedures were modified Blalock–Taussig shunt (65.8%) and total cavopulmonary connection with an extracardiac conduit fenestrated without cardiopulmonary bypass (15.9%). We did not find any factors associated with negative outcomes in these patients. The overall survival was 86.8%, with a better outcome in those who did not require reintubation (log rank, *p* < 0.01). The survival of RAI was similar to other centers. Individuals with RAI should be evaluated rigorously to determine an adequate repair strategy, considering high morbidity and mortality.

## 1. Introduction

During the embryonic growth of vertebrates, the heart is the first organ to be developed through cardiogenesis, a specialized process that involves various interactions between morphogenetic and transcriptional pathways. Any deregulation that impacts the expression of cardiac genes could affect the development of the heart and, therefore, cause cardiac malformations [1,2].

Globally, cardiac malformations in live newborns reach 0.8%. Although these malformations are likely inherited, up until now, their origin has not been exactly defined, and they are considered to be of a multifactorial cause [3].

The prevalence varies in each region, ranging from 2.1 to 12.3 per 1000 live births [4]^,^ with an incidence of 6 to 8 per 1000 live births worldwide [5], where atrial isomerism (AI) is one of the most serious and least frequent forms, with a prevalence of 1 in every 10,000 to 20,000 live births worldwide [6,7].

AI is a heart malformation of the body’s left–right axis, having mirror images symmetrical to each other, with normal morphology of the left–right side [3,7,8,9], resulting in the impossibility of establishing normal left–right asymmetry during the embryonic development [10]. Taking into consideration the functional relevance of organ asymmetry in humans, the heart is undoubtedly the most striking case. The heart not only has an asymmetric position within the thorax, but it is also asymmetrically constructed. Both the left atrium and right atrium, as well as the left ventricle and right ventricle, differ in several aspects, including their pumping performance and connections to arteries and veins [10].

Laterality problems come with high uncertainty due to limited knowledge and the impossibility of accurately determine the existing situation, as well as the possibility of more than one outcome. Various investigations have shown that more than 80 genes are involved in the development of normal asymmetric organs. Mutations in a few genes have been identified in patients with laterality disorders, such as *Nodal* and *Pitx2* genes, as well as *NKX2.5*, *CRELD1*, *LEFTY2*, *ZIC3* and *CRIPTC* genes, which are associated with the encoding of components involved in the transforming growth factor beta (TGF-β) pathway. When the TGF-β pathway is altered, it causes one of two entities: right atrial isomerism (RAI) or left atrial isomerism (LAI) [3,11,12].

RAI is typically associated with complex cardiovascular malformations [7], and given its infrequent presentation, both diagnosis and medical/surgical management involve several methods and alternatives [13,14,15,16], from palliative surgery through univentricular physiology to total correction surgery for biventricular repair [7,8]. The postoperative mortality is high in these patients; furthermore, it increases when risk factors are present, such as valvular regurgitation and total anomalous pulmonary vein connection (TAPVC), among others [7,8]. TAPVC represents a surgical emergency in congenital heart surgery; therefore, achieving accurate surgery with minimal damage to the myocardium is vital. TAPVC is strongly associated with RAI [17,18].

As far as we know, the epidemiological impact of RAI on identifying determinants of increased morbidity and mortality has yet to be studied in Mexican patients. In this regard, this research represents the importance of early detection and prevention of this complex disease. Therefore, the aim of this study was to analyze the mortality and surgical outcomes of patients with RAI who underwent cardiac surgery. 

## 2. Materials and Methods

### 2.1. Study Population

The study was approved by the local institutional review board (CI-006-2023) and the need for informed consent was waived. We performed an observational, descriptive and retrospective study that included patients under 18 years of age, diagnosed with RAI, who underwent cardiac surgery and were followed-up from 1 January 2010 to 31 March 2020. Patients who underwent surgery at a different hospital or had incomplete medical records were excluded from this study. The variables of interest were collected from the electronic medical records and surgical notes. Demographic data, primary diagnoses, previous interventions, including cardiac surgery and cardiac catheterization, and chest X-ray, echocardiographic, computed tomography and magnetic resonance imaging data were recorded. Surgical variables included date of admission, date of surgery, indication for surgery, details of the cardiac morphology, morbidity and any complications prior to discharge.

The diagnosis of RAI was determined through the assessment of morphological features, considering a morphologically right atrium when the appendage was pyramidal, the crista terminalis was evident and the pectineal muscles were extending towards the vestibule of the tricuspid valve in its entire parietal extension. Additionally, the bronchopulmonary morphology was assessed, where the right bronchus was more horizontal and shorter than the left bronchus, with three lobes, and the right pulmonary branch was crossing anterior and slightly inferior to it [19]. The diagnosis of RAI was supported with echocardiography, computed tomography, magnetic resonance imaging and cardiac catheterization studies. 

Arterial hypotension was considered when the systolic blood pressure was below the 5th percentile according to age [20]. Vascular injury was defined as eventual vessel damage that may occur before the surgical procedure. Major bleeding was defined as blood loss of 7 mL/kg/h or more in 2 or more consecutive hours in the first 12 postoperative hours, or 84 mL/kg or more during the first 24 postoperative hours, or when a surgical re-exploration was needed due to hemorrhage during the first 24 postoperative hours [21]. 

The follow-up of patients was held in the outpatient clinic at 1, 3, 6 and 12 months after surgery; then, the follow-ups continued annually, unless an earlier follow-up was necessary. Every follow-up stage was documented, up to the last visit in March 2020. Early mortality was defined as death occurring during the first 30 days after surgery.

### 2.2. Statistical Analysis

Descriptive statistics were calculated to analyze demographic variables. To describe the categorical variables, frequencies and percentages (%) were used, while quantitative variables were represented in median values, interquartile ranges (IQRs), means and standard deviations (±SDs). Quantitative variables’ normal distribution was tested using the Kolmogorov–Smirnov test. The chi-square test was used to compare proportions; however, when fewer than five observations were made, the double-ended Fisher’s exact test was used. Patient survival was analyzed using the Kaplan–Meier method and compared using the log-rank test. A *p* value < 0.05 was considered statistically significant. The software used was SPSS version 24.0 SPSS Inc., Chicago, IL, USA.

## 3. Results

### 3.1. Demographic Characteristics

We included 38 patients with RAI; 57.9% (n = 22) were men. The median age was 4 years (IQR 2–9.2), the median weight was 14.3 kg (IQR 9.8–22.1) and the mean height was 102.2 ± 29.1 cm (Table 1). Univentricular morphology was found in every patient. Dextrocardia was found in 44.7% (n = 17), and 31.6% (n = 12) of patients had had at least one previous operation. An association with TAPVC was observed in 34.3% (n = 13), all of whom presented the supracardiac variety. Stenosis in one of the pulmonary artery branches was found in 60.5% (n = 23). The most frequent degree of atrioventricular valve regurgitation (Table 1) was mild, found in 71% (n = 27), while the severe degree was found in 5.3% (n = 2).

The main diagnoses associated were atrioventricular septal defect (n = 24; 63.2%) and pulmonary stenosis (n = 21; 55.3%) (Table 2). During the preoperative period, 13.2% (n = 5) of patients required support with inotrope use and 15.9% (n = 6) of patients required intubation; no patient presented preoperative infection.

### 3.2. Surgical Characteristics

The main surgical procedures (Table 2) performed were modified Blalock–Taussig shunt (MBTS) (n = 25; 65.8%) and total cavopulmonary connection (TCPC) with an extracardiac conduit fenestrated without cardiopulmonary bypass (n = 6; 15.9%). 

Twelve (31.6%) patients had at least one previous surgery. Of those, 11 (28.9%) had an MBTS, of which in 7 (18.4%) cases, a new MBTS was placed, and in the other 4 (10.5%) cases, a bidirectional cavopulmonary connection (BCPC) was performed. One (2.6%) case with previous BCPC underwent a TCPC with an extracardiac conduit fenestrated. 

In 11 (28.9%) cases, the MBTS was associated with the repair of the TAPVC; in 1 (2.6%) case, the MBTS was associated with an atrioventricular valve replacement as well as TAPVC repair (Table 2). The univentricular repair strategy was used in all cases. Four (10.5%) patients who required emergency surgery were identified; in all of them, MBTS was performed.

### 3.3. Early Results

Of the 38 patients included, 13.2% (n = 5) died after cardiogenic shock. The main transoperative complications were arterial hypotension (n = 13; 34.3%), cardiorespiratory arrest (n = 2; 5.3%), supraventricular tachycardia (n = 2; 5.3%), ventricular fibrillation (n = 1, 2.6%), atrioventricular block (n = 1, 2.6%), vascular injury (n = 1; 2.6%) and pulmonary hypertension (n = 1; 2.6%).

Fifteen (39.5%) surgeries were performed with cardiopulmonary bypass (Table 3), with a mean time of 95.4 ± 33.7 min; the mean aortic cross-clamp time was 53.3 ± 30.5 min. 

The median length of stay in the pediatric intensive care unit was 4 days (IQR 2.2–7.7), and the median duration of mechanical ventilation was 22 hours (IQR 10–126) (Table 3). The postoperative complications were heart failure (n = 23; 60.5%), pleural effusion (n = 13; 34.3%), pulmonary hypertension (mild n = 2, 5.3%; moderate n = 3, 7.9%), infectious processes (n = 5; 13.2%), supraventricular tachycardia (n = 2; 5.3%), atrioventricular block (n = 1; 2.6%), major bleeding (n = 2; 5.3%) and pneumothorax (n = 2; 5.3%). Four (10.5%) patients required inhaled nitric oxide (iNO).

Five patients (13.2%) underwent a new surgical intervention: two (5.3%) cases due to major bleeding, two (5.3%) due to surgical site infection and one (2.6%) due to takedown of BCPC. Additionally, 21.1% (n = 8) of patients required cardiac catheterization after surgery: angioplasty with stent in pulmonary branches (n = 2; 5.3%), placement of a stent in the fenestration of the extracardiac conduit (n = 2; 5.3%), angioplasty of the MBTS (n = 1; 2.6%) and closure of the main pulmonary artery (n = 1; 2.6%). The last two (5.3%) patients did not need additional therapeutic support.

### 3.4. Follow-Up

A postoperative follow-up of 117 ± 4.1 months was conducted. We found no statistically significant differences between patients who survived the surgery and those who died in terms of their age, weight, height, prior surgery, cardiopulmonary bypass time, aortic cross-clamp time, mechanical ventilation and length of stay in the intensive care unit, among other factors (Table 4). In addition, there were no factors associated with mortality in the multivariate analysis.

**Figure 1:**
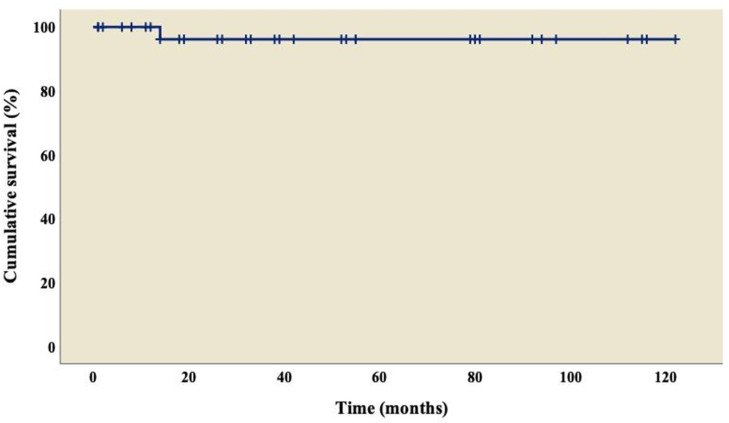
Overall survival curve of patients with right atrial isomerism.

**Figure 2:**
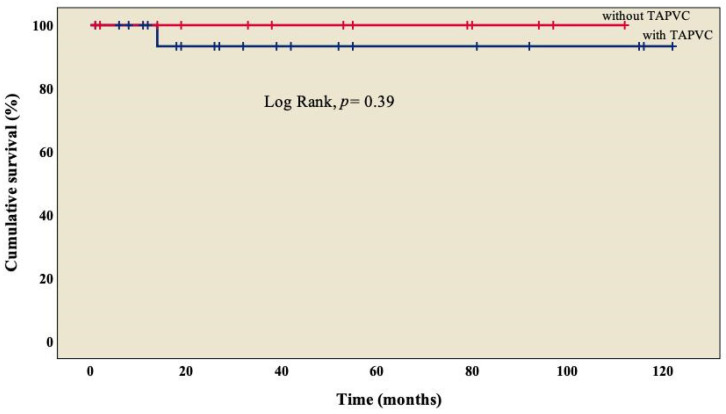
Kaplan–Meier curves of patients with right atrial isomerism, with and without TAPVC. TAPVC: total anomalous pulmonary venous connection.

**Figure 3:**
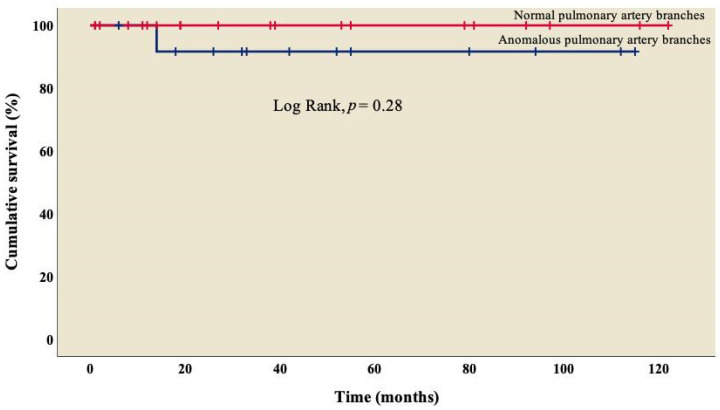
Kaplan–Meier curves of patients with right atrial isomerism, with and without anomalous in pulmonary artery branches.

The analysis showed an overall survival of 86.8% at 10 years of follow-up, which became constant from the 18th month after surgery (Figure 1). A better outcome was observed in patients who did not present TAPVC (Figure 2) or anomalies in the pulmonary branches (Figure 3); from the 18th month of follow-up, those without TAPVC achieved a survival rate of 95%, while those without anomalies in the pulmonary branches achieved a survival rate of 92.3%.

**Figure 4:**
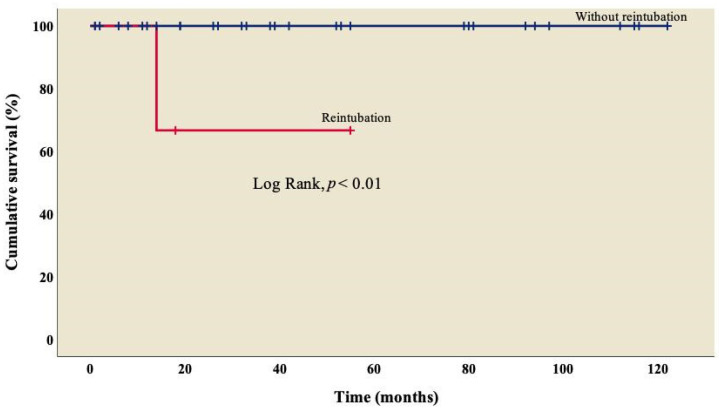
Kaplan–Meier curves of patients with right atrial isomerism, with and without reintubation.

Finally, survival was lower (66.7%) in patients who required reintubation when compared to those who did not (Figure 4); this difference was statistically significant (log rank, *p* < 0.01).

## 4. Discussion

AI is a complex entity associated with various malformations, observed in both cardiovascular and other systems [6,7,22]; in addition to being a rare condition, according to the Society of Thoracic Surgeons [23], AI represents 1.95% of surgical procedures in patients with congenital heart disease.

Patients with cardiac malformations used to be considered inoperable, but this has been changing in recent years. However, the treatment of heterotaxy syndrome is evidently challenging due to the low survival in the short and medium terms. The above was confirmed in a report provided by the Hospital for Sick Children, which stated that in a series of 91 patients with RAI who were followed up for over a 26-year period, the overall mortality was 69%, while the overall survival estimates were 71% at 1 month, 49% at 1 year and 35% at 5 years [24].

Observations in different populations of patients with RAI who underwent univentricular or biventricular repair have been relatively scarce; however, an encouraging picture has emerged in recent years. For instance, the predominant form is RAI [7,22,25,26,27,28,29,30,31,32], which is found in 58.5% of patients with AI in our center. According to Baban et al. [25] and Alongi et al. [26], the diagnosis is always a challenge, even when we can directly visualize the atrial morphology during the surgical procedure. However, in our center, the 38 patients included in this study were diagnosed in the preoperative stage with the support of auxiliary diagnostic methods. The initial evaluation in the neonatal stage is fundamental, as well as early postnatal care [33], due to the fact that the persistence of the right umbilical vein has recently been identified in 42% of cases of AI in the fetal period, predominantly in RAI, with 73% [34], and should include the coordinated actions of teams with different specialties, not only pediatric cardiology and pediatric cardiac surgery.

We observed TAPVC in 34.3% (n = 13) of patients (Table 1), similar to what has been marked by other groups, with a survival rate of 95% (Figure 2), without a significant difference from those who did not present TAPVC (log rank, *p* = 0.39) [22,25,26]. Not a single patient presented obstruction of the pulmonary venous system, which differs from what was observed by other groups, where it is found in between 9% and 25% of cases [18,31]; this condition is considered a factor of morbidity and mortality [26,29,33], in agreement with Chen et al. (OR: 44.338, *p* = 0.005) [22] and Alongi et al. (HR: 4.40, *p* = 0.010) [26]. At this point, we must mention that in RAI, it is common to have two morphological right atriums that, when arriving at the spatially located left atrium (but morphologically a right atrium), result in an abnormal pulmonary venous connection, which is observed in every patient.

We agree that pulmonary venous return anomalies are the norm in patients with RAI and consequent secondary pulmonary hypertension; however, there are pulmonary alterations that go beyond pulmonary vascular alterations. The close relationship between morphological alterations in laterality and the presence of primary ciliary dyskinesia (PCD), as well as the indispensable role of ciliary function in the embryonic node for proper differentiation in left–right laterality, has led to the search for a genetic origin linking both entities [35,36]. Thus, Nakhle et al. [37] found that 42% of patients with congenital heart disease associated with AI have some degree of ciliary dysfunction. In a retrospective study by Kennedy et al. [36], 76% of cases presented with neonatal respiratory distress and 100% of patients older than 18 years had a history of bronchiectasis. These characteristics of ciliary motility patterns and alterations in the pulmonary vasculature in patients with RAI mean that postoperative mechanical ventilation in these patients is prolonged in up to 20% of cases [38].

On the other hand, 60.5% (n = 23) of patients had some degree of stenosis in one of the pulmonary artery branches (Table 1), with a survival rate of 92.3% (Figure 3) and no significant difference from those who did not present it (log rank, *p* = 0.28); similarly, in the univariate analysis, we observed that stenosis was not associated with mortality (*p* = 0.31), which coincides with what was reported by McGovern et al., where atresia in one of the pulmonary artery branches was present in 50% of cases, but it was not related to mortality in this group (*p* = 0.37) [39].

Notably, 30 (78.9%) patients had some degree of valvular regurgitation, of which 2.6% (n = 1) was moderate, and 5.3% (n = 2) was severe. Valvular regurgitation has been identified as a factor that contributes to mortality in some studies [26,39]. However, due to the limited number of patients in our investigation, we do not have statistical evidence to suggest that valvular regurgitation is associated with negative clinical outcomes. 

The overall survival at 10-year follow-up reached 86.8% (Figure 1), which is higher than in other reports, ranging from 44% to 70% [8,22,25,26,31,39]. Given the wide variety of cardiac malformations in AI, choosing the best repair strategies is a real challenge; we can highlight that our group used a univentricular strategy in all the patients, where the main procedure was MBTS (n= 25; 65.8%), followed by TCPC with an extracardiac conduit fenestrated (n = 6; 15.9%) and BCPC without cardiopulmonary bypass (n = 3; 7.9%), similar to what was reported by Alongi et al. [26]. We must emphasize that the objective of the treatment in these patients is to reduce the volume overload in the only functional ventricle. In this way, the surgery seeks to decongest this workload progressively and at some point, if possible, to separate the two circuits, pulmonary and systemic, going from a parallel circulation into a serial circulation. Palliative procedures are diverse and are based on the anatomical variants of this pathology; therefore, surgery can be performed to create a systemic pulmonary shunt when there is obstruction in the pulmonary circulation or perform pulmonary artery banding in cases of pulmonary overcirculation [40,41].

In addition to the surgical procedure, cardiac catheterization, the rate of which reached 21.1% in our center, is an important complementary method, especially for a description of the vascular anatomy; furthermore, catheterization improves the physiological response of the pulmonary vasculature in older individuals with a structural heart disease, and in cases of suspected obstruction related to the pulmonary venous systems [19]. 

We assessed the severity of the patient’s illness based on their use of inotropes and intubation status. Inotropic use as hemodynamic support during the pre-, trans- and postoperative stages was not associated with mortality. Additionally, no association was observed between preoperative intubation status and mortality. 

Patients with RAI have dual sinus nodes and dual atrioventricular nodes, which makes them more susceptible to supraventricular tachycardia as a result of the severely impacted atrial topology [42,43]. Our study discovered that up to 5.3% of patients with this condition experience this phenomenon without any negative hemodynamic effects and are currently being monitored.

Instead, the absence of spleen has been considered a complementary part of the diagnosis of RAI. Recent reports indicate various varieties of spleen presentations [25,26,29,39], finding asplenia in 68% to 79% of patients with RAI, so the majority of infectious processes caused by encapsulated bacteria mainly occurs in these patients [25,29,33,39]. Therefore, Bhaskar et al. (HR: 2, *p* = 0.008) [29] and Banka et al. (HR: 1.67, *p* = 0.044) [33] have considered it as a predictor of mortality. However, there were no patients with infectious processes in our center in the preoperative stage.

It is important to take into account three scenarios at the time of diagnosis: first, the predominance of RAI in patients with AI; second, despite an all-in-one diagnostic approach, a precise diagnosis is sometimes challenging, even with a direct visualization of the atrial morphology during the surgical event; and third, patients with AI, mainly RAI, show an association with other extracardiac alterations, so complementary diagnostic approaches such as abdominal ultrasonography and contrast-enhanced imaging studies at the gastrointestinal level should be considered based on the findings during the initial evaluation, in addition to the multidisciplinary work in the care of these patients [19].

We acknowledge the limitations of our study. First, the typical retrospective, single-center, non-randomized study limits the generalizability of our findings. Second, the small sample size makes it challenging to identify risk factors for mortality since the statistical power to discern differences is relatively low. We collected a comprehensive set of variables to evaluate, but there may be others we should have measured that could have influenced our outcome.

Additionally, given the limited sample size, it is imperative to conduct further studies with larger sample sizes that include more patients to enhance the reliability and consistency of the study results. Nevertheless, previous reports in the literature have also featured small sample sizes (35–70 patients) owing to the infrequent occurrence of the disease [18,22,31,39]. Our cohort offers valuable insight into the short- and long-term health of these patients, not only within our medical center but throughout the entire region and country. This information aids in the development of preliminary prognostic factors that can be modified, implemented and supplemented with new therapeutic options.

In summary, RAI is considered one of the most severe forms of congenital heart disease. However, our research demonstrates that patients at our referral center had a comparable survival rate to other centers. Although we evaluated the most frequently described factors related to mortality, including age, anomalous pulmonary venous connection and atrioventricular valve regurgitation, we did not find that our patients with the abovementioned characteristics were more likely to die. However, caution should be taken when interpreting these outcomes in populations with a higher prevalence of this condition. Therefore, a comprehensive assessment is imperative for an accurate diagnosis, which is crucial in identifying the most effective treatment approach with an appropriate repair plan, while taking into account the associated morbidity and mortality.

## Figures and Tables

**Table 1:** Overall characteristics of patient with right atrial isomerism.

Characteristics	Total n = 38
Sex, n (%)	
Male	22 (57.9)
Female	16 (42.1)
Age (years), median (IQR)	4 (2–9.2)
Weight (kg), median (IQR)	14.3 (9.8–22.1)
Height (cm), mean (±SD)	102 (29.1)
Previous surgery, n (%)	
0	25 (65.8)
1	12 (31.6)
2	1 (2.6)
RACHS-1, n (%)	
2	5 (13.2)
3	33 (86.8)
Cardiac intrathoracic position, n (%)	
Levocardia	21 (55.3)
Dextrocardia	17 (44.7)
Atrioventricular valve regurgitation, n (%)	
None	8 (21.1)
Mild	27 (71)
Moderate	1 (2.6)
Severe	2 (5.3)
Anomalous pulmonary venous connection, n (%)	
Partial	2 (5.3)
Total	13 (34.3)
Pulmonary venous connection, n (%)	
Right atrium	15 (39.5)
Left atrium	20 (52.6)
Both atriums	3 (7.9)
Pulmonary artery branches, n (%)	
Normal	15 (39.5)
Stenosis	23 (60.5)

IQR: interquartile range, RACHS-1: Risk Adjustment for Congenital Heart Surgery 1, SD: standard deviation.

**Table 2:** Associated defects and cardiac surgeries performed in patients with right atrial isomerism.

Characteristic, n (%)	Total n = 38
Diagnosis	
AV canal	24 (63.2)
Pulmonary stenosis	21 (55.3)
PDA	17 (44.7)
Pulmonary atresia	15 (39.5)
TAPVC	13 (34.3)
DORV	9 (23.7)
Hypoplastic left ventricle	6 (15.9)
PAPVC	2 (5.3)
Surgery	
MBTS	13 (34.3)
MBTS + TAPVC repair	10 (26.3)
TCPC with an extracardiac conduit fenestrated without CPB	6 (15.9)
BCPC without CPB	3 (7.9)
MBTS + thrombectomy of pulmonary artery + TAPVC repair	1 (2.6)
BCPC with CPB + TAPVC repair	1 (2.6)
BCPC with CPB + RPA angioplasty + PAPVC repair	1 (2.6)
TCPC with an extracardiac conduit fenestrated + PAPVC repair	1 (2.6)
Mechanical AV valve replacement + MBTS + TAPVC repair	1 (2.6)
BCPC takedown + MBTS	1 (2.6)

AV: atrioventricular, BCPC: bidirectional cavopulmonary connection, CPB: cardiopulmonary bypass, DORV: double-outlet right ventricle, MBTS: modified Blalock–Taussig shunt, PAPVC: partial anomalous pulmonary venous connection, PDA: patent ductus arteriosus, RPA: right pulmonary artery, TAPVC: total anomalous pulmonary venous connection, TCPC: total cavopulmonary connection.

**Table 3:** Operative and postoperative characteristics of patients with atrial isomerism.

Characteristic	Total n = 38
Surgery with CPB, n (%)	15 (39.5)
CPB (min), median (IQR)	89 (67–107)
Aortic cross-clamp (min), median (IQR)	55 (22–83)
Mechanical ventilation in PICU, n (%)	34 (89.5)
Mechanical ventilation time (h), median (IQR)	22 (10–126)
Reintubation, n (%)	3 (7.9)
Days in PICU, n (%)	
<1 day	2 (5.3)
1–7 days	27 (71)
8–15 days	8 (21.1)
>15 days	1 (2.6)

CPB: cardiopulmonary bypass, IQR: interquartile range, PICU: pediatric intensive care unit.

**Table 4:** Risk factors associated with mortality (univariate analysis).

Variable	Alive		Dead		OR	CI 95%		*p*
	n	%	n	%		Lower	Upper	
Previous surgery	11	33.3	2	40	1.33	0.19	9.18	1
Anomalous pulmonary venous connection	13	39.4	0	0	-	-	-	-
Anomalous pulmonary artery branches	21	63.6	2	40	0.38	0.06	2.61	0.37
Atrioventricular valve regurgitation	25	81.8	3	60	0.33	0.05	2.46	0.28
Atrioventricular valve replacement	0	0	1	20	1.25	0.81	1.94	0.13
Preoperative intubation	4	12.1	2	40	4.83	0.61	38.39	0.17
Preoperative inotrope use	3	9.1	2	40	6.67	0.78	57.06	0.12
Transoperative inotrope use	26	78.8	5	100	0.78	0.66	0.94	0.56
Postoperative inotrope use	28	84.8	5	100	0.84	0.74	0.98	1
Inhaled nitric oxide use	2	6.1	2	40	10.33	1.05	102.08	0.08

CI: confidence interval, OR: odds ratio.

## Data Availability

Data supporting the results are available from the corresponding authors upon reasonable request.
